# Prognostic Value of Post First-Line Chemotherapy Glasgow Prognostic Score in Advanced Non-Small Cell Lung Cancer

**DOI:** 10.1177/11795549221086578

**Published:** 2022-03-22

**Authors:** Kristin Stokke, Marie Søfteland Sandvei, Bjørn Henning Grønberg, Marit Slaaen, Kristin T Killingberg, Tarje O Halvorsen

**Affiliations:** 1 Department of Clinical and Molecular Medicine, Norwegian University of Science and Technology (NTNU), Trondheim, Norway; 2 The Cancer Clinic, St. Olavs Hospital, Trondheim University Hospital, Trondheim, Norway; 3 Department of Public Health and Nursing, Norwegian University of Science and Technology (NTNU), Trondheim, Norway; 4 Research Centre for Age-related Functional Decline and Disease (AFS), Innlandet Hospital Trust HF, Hamar, Norway; 5 Institute of Clinical Medicine, University of Oslo, Oslo, Norway

**Keywords:** Lung cancer, non-small cell lung cancer (NSCLC), prognostic biomarker, chemotherapy, maintenance therapy

## Abstract

**Background:**

The Glasgow prognostic score (GPS) is an established inflammatory prognostic index in cancer patients. Most studies have only measured GPS at baseline (B-GPS). Effective cancer therapy may reduce inflammation, and we investigated whether re-assessing GPS after first-line chemotherapy (E-GPS) provided more prognostic information than B-GPS in a phase III trial of advanced non-squamous non-small cell lung cancer (NSCLC).

**Methods:**

Glasgow prognostic score was assessed before and after carboplatin/vinorelbine chemotherapy. When assessing GPS, C-reactive protein (CRP) ⩾ 10 mg/L and albumin < 35 mg/L are defined as abnormal values. GPS 0: both values normal, GPS 1: one abnormal value, and GPS 2: both values abnormal.

**Results:**

Glasgow prognostic score at baseline and E-GPS were available in 138 patients. Median age was 67 years, 51% were women, and 94% had performance status 0-1. B-GPS was not a statistically significant prognostic factor (B-GPS 1 vs 0: hazard ratio [HR] = 1.32, 95% confidence interval [CI] = 0.9-2.0; B-GPS 2 vs 0: HR = 1.46, 95% CI = 0.9-2.3), while E-GPS was (E-GPS 1 vs 0: HR = 1.57, 95% CI = 1.0-2.4; E-GPS 2 vs 0: HR = 2.77, 95% CI = 1.7-4.5). E-GPS was associated with treatment response (*P* < .01), whereas B-GPS was not.

**Conclusion:**

Glasgow prognostic score at baseline after first-line chemotherapy provided more prognostic information than baseline GPS in patients with advanced non-squamous NSCLC and was associated with treatment response.

**ClinicalTrials.gov Identifier:**

NCT02004184.

## Background

Inflammation plays an essential role in cancer development and progression,^1-3^ and the development and maintenance of a systemic inflammatory response has been consistently associated with poorer outcome in both early and advanced disease.^4^

Glasgow prognostic score (GPS) is an inflammatory score based on values of C-reactive protein (CRP) and albumin.^5^ Mounting evidence has shown that it is an independent prognostic factor in numerous cancers, different disease stages, and treatment settings.^6-13^ An important aspect is that it is objectively assessed, affordable, and easy to implement in clinical practice.

Almost all previous studies have measured GPS only once and mainly before start of treatment (at baseline, “B-GPS”).^5-9,11-23^ However, GPS is believed to reflect inflammation as an expression of cancer activity, and hence, in patients who respond to cancer treatment, a reduction in inflammation and thereby in GPS is to be expected.^4^ Thus, GPS measured after treatment (at evaluation, “E-GPS”) might capture the effect of treatment and be a more precise prognostic factor than B-GPS.

Lung cancer is marked by high inflammation and poor survival,^24,25^ and a high proportion of patients have elevated GPS as compared with other cancer types.^25^ Therefore, in a randomized phase III trial comparing immediate maintenance pemetrexed with pemetrexed at progression in patients with advanced non-squamous non-small cell lung cancer (NSCLC),^26^ we measured B-GPS and E-GPS after induction chemotherapy. The aims were to assess whether E-GPS provides better prognostic information than B-GPS and whether there were associations between response to chemotherapy and B-GPS, E-GPS, or change in GPS.

## Methods

### Approvals

This open randomized phase III multicenter trial was approved by the Regional Committee for Medical Research Ethics in Central Norway (ID 2013/645, approved on June 17, 2013) and The Norwegian Medicines Agency.

### Patients

From May 2014 to September 2017, a total of 232 patients were enrolled at 19 hospitals in Norway. Eligible patients were treatment naïve, had stage IIIB-IV non-squamous NSCLC, no known activating EGFR-mutation or ALK-translocation, WHO performance status (PS) 0-2, and adequate bone marrow/liver/kidney function. Patient were to receive 4 courses of induction chemotherapy with carboplatin AUC 5 (Calvert’s formula) IV and vinorelbine 25 mg/m^2^ IV day 1 and vinorelbine 25 mg/m^2^ IV or 60 mg/m^2^ PO day 8, every 3 weeks. Patients who completed 4 courses, had PS 0-2 and non-progression were randomized to immediate maintenance pemetrexed therapy or observation. Pemetrexed was the treatment of choice at progression. Patients who were not randomized were treated according to each hospital’s routines. The study closed prematurely when immunotherapy became available in Norway and replaced pemetrexed as standard relapse treatment. In the randomized trial, there was no significant difference in overall survival (OS) (*P* = .10) between treatment arms. Thus, in the present study, all patients were analyzed as one cohort.^26^

**Figure 1.**
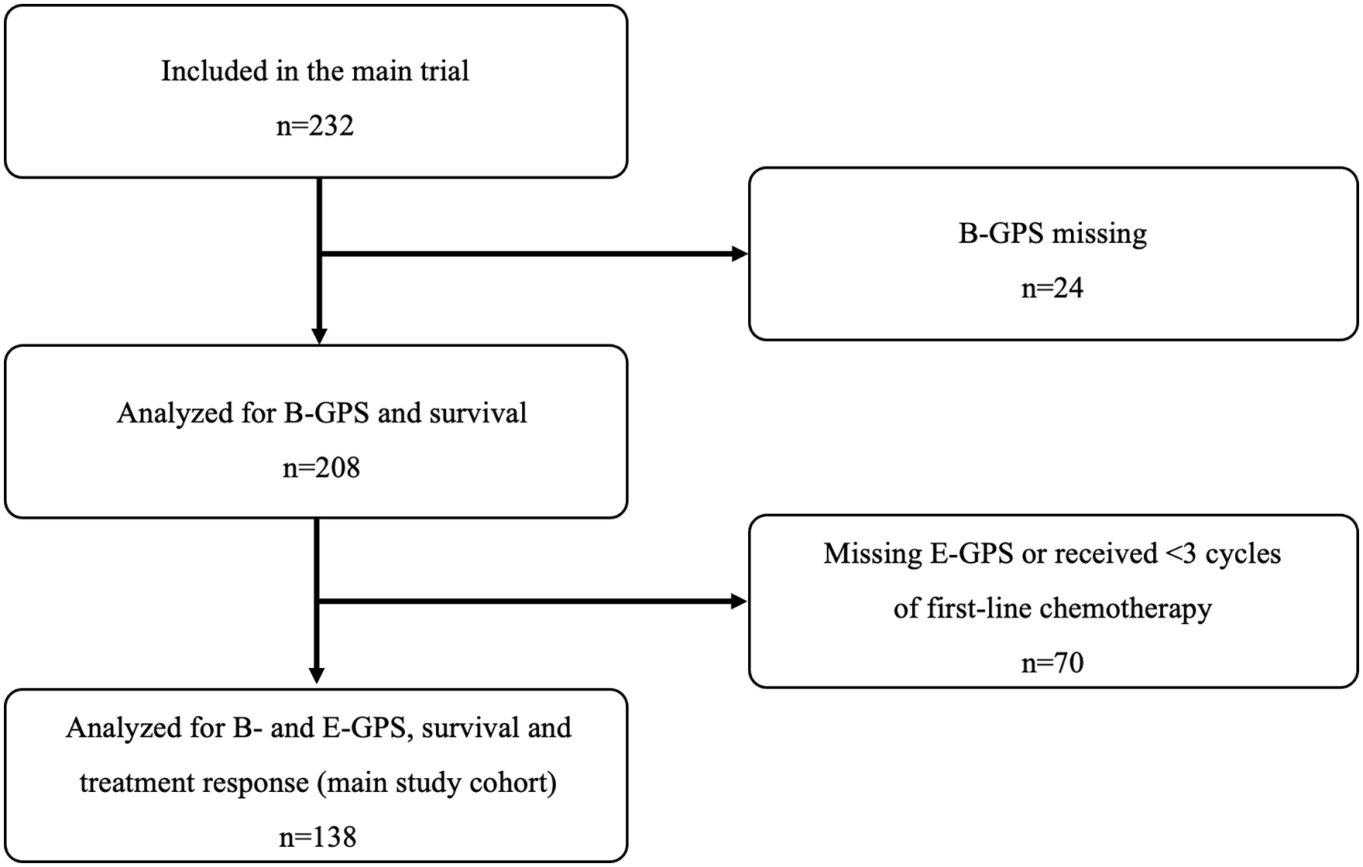
Consort flow diagram.

For our main analyses, we included patients who received 3 or 4 courses of carboplatin/vinorelbine if GPS was scored both at baseline and evaluation (main study cohort) (Figure 1). In a sensitivity analysis of B-GPS and survival, we included all patients with a B-GPS, independent of number of completed chemotherapy courses (Figure 1).

### Glasgow prognostic score

According to the GPS, an elevated CRP of ⩾10 mg/L and hypoalbuminemia of <35 mg/L are considered abnormal values. If both values are normal, GPS is 0. If one value is abnormal, GPS is 1, and when both are abnormal, GPS is 2. A higher score is associated with shorter survival.^5^

Blood samples for assessing GPS were collected within 2 weeks before chemotherapy commenced (B-GPS) and within 3 weeks after the last chemotherapy course was administered (E-GPS).

### Endpoints

Overall survival was defined as time from inclusion until death of any cause in the analyses with B-GPS and as time from evaluation after first-line chemotherapy until death of any cause in analyses with E-GPS. Response to treatment was assessed according to the Response Evaluation Criteria in Solid Tumors (RECIST) version 1.1.^27^

### Statistical considerations

Survival was estimated using the Kaplan-Meier method and compared using the Cox proportional hazard model. To assess the prognostic value of B-GPS and E-GPS in our main study cohort, multivariable Cox proportional hazards models for survival were adjusted for sex, age (continuous variable), and stage of disease (III vs IV). In addition, we adjusted for PS scored at baseline when examining B-GPS, and PS at evaluation in models with E-GPS. Performance status at evaluation was missing for 6 patients who were excluded from multivariable analyses.

As E-GPS was not measured in all patients with a B-GPS, we performed sensitivity survival analyses including all patients with a B-GPS (n = 208) to account for a potential selection bias.

Finally, we performed sensitivity survival analyses in the main study cohort (n = 138), adjusting for randomization (no/observation-arm/maintenance-arm), and whether patients received immunotherapy after chemotherapy, as this has been shown to significantly improve survival in some patients with advanced NSCLC.^28,29^

Associations between B-/E-GPS and response to chemotherapy were compared using Pearson’s chi-square test or Fisher’s exact test. A 2-sided *P* < .05 was considered statistically significant. SPSS Version 27.0 (Armonk, NY: IBM Corp) was used for all statistical analyses.

## Results

### Patients

For 138 (59%) of the 232 patients enrolled in the randomized controlled trial (RCT), both B-GPS and E-GPS were available. These patients were included in the present study as our main study cohort. B-GPS was measured in an additional 70 patients, who were also included in sensitivity analyses, whereas 24 patients had no GPS measures and were excluded altogether (Figure 1).

In our main study cohort, median age was 67 years (range, 47-83), 70 (51%) were women, 130 (94%) had stage IV disease, and 50 (36%), 80 (58%), and 8 (6%) had PS 0, 1, and 2, respectively (Table 1). After completing induction chemotherapy, 80 (58%) of the patients were randomized to immediate maintenance pemetrexed therapy (n = 42) or observation (n = 38). Thirty-six (26%) of the patients received immunotherapy after the study therapy (Table 1). Mean follow-up time was 14.1 months (95% confidence interval [CI] = 12.3-15.9). Eighteen of 138 patients were alive when follow-up was completed.

**Table 1.** Patient characteristics of all patients in the main study cohort.

		Main study cohort	GPS at baseline (B-GPS)			GPS at evaluation (E-GPS)		
		n = 138	B-GPS 0 n = 55	B-GPS 1 n = 53	B-GPS 2 n = 30	E-GPS 0 n = 59	E-GPS 1 n = 50	E-GPS 2 n = 29
		n (%)	n (%)	n (%)	n (%)	n (%)	n (%)	n (%)
Age	Median (range)	67 (47-83)	65 (47-83)	68 (56-81)	66 (50-82)	65 (47-81)	68 (51-83)	66 (50-77)
Sex	Male	68 (49)	30 (55)	24 (45)	14 (47)	26 (44)	25 (50)	17 (59)
	Female	70 (51)	25 (45)	29 (55)	16 (53)	33 (56)	25 (50)	12 (41)
Stage	IIIb	8 (6)	3 (5)	3 (6)	2 (7)	6 (10)	2 (4)	–
	IV	130 (94)	52 (95)	50 (94)	28 (93)	53 (90)	48 (96)	29 (100)
WHO PS	0	50 (36)	28 (51)	16 (30)	6 (20)	26 (44)	18 (36)	6 (21)
	1	80 (58)	24 (44)	33 (62)	23 (77)	30 (51)	29 (58)	21 (72)
	2	8 (6)	3 (5)	4 (8)	1 (3)	3 (5)	3 (6)	2 (7)
Randomization	No	58 (42)	21 (38)	25 (47)	12 (40)	17 (29)	22 (44)	19 (66)
	Observation	38 (28)	18 (33)	10 (19)	10 (33)	22 (38)	13 (26)	3 (10)
	Maintenance	42 (30)	16 (29)	18 (34)	8 (27)	20 (34)	15 (30)	7 (24)
Received immunotherapy after chemotherapy	No	102 (74)	37 (67)	43 (81)	22 (73)	38 (64)	40 (80)	24 (83)
	Yes	36 (26)	18 (33)	10 (19)	8 (27)	21 (36)	10 (20)	5 (17)

GPS, Glasgow Prognostic Score; WHO PS, WHO Performance Status.

In our main study cohort, 55 (40%) patients had B-GPS 0, 53 (38%) B-GPS 1, and 30 (22%) B-GPS 2. At evaluation after induction chemotherapy, 59 (43%) patients had E-GPS 0, 50 (36%) E-GPS 1, and 29 (21%) E-GPS 2 (Table 1). Patients with B-GPS 0 were more likely to have PS 0 than patients with B-GPS 1-2. Otherwise, baseline and treatment characteristics were balanced between patients with B-GPS 0, 1, and 2.

Seventy-three patients (53%) had no change in GPS. Thirty-three patients (24%) improved their GPS; 19 (14%) from 1 to 0, 8 (6%) from 2 to 1, and 6 (4%) from 2 to 0. Glasgow prognostic score deteriorated in 32 (23%) patients; 19 (14%) from 0 to 1, 2 (1%) from 0 to 2, and 11 (8%) from 1 to 2.

Baseline characteristics of the 70 patients included in the sensitivity analysis and the 24 excluded patients (Figure 1) were comparable to the characteristics of the main study cohort (Supplementary Table).

### Overall survival

#### B-GPS and survival

**Figure 2.**
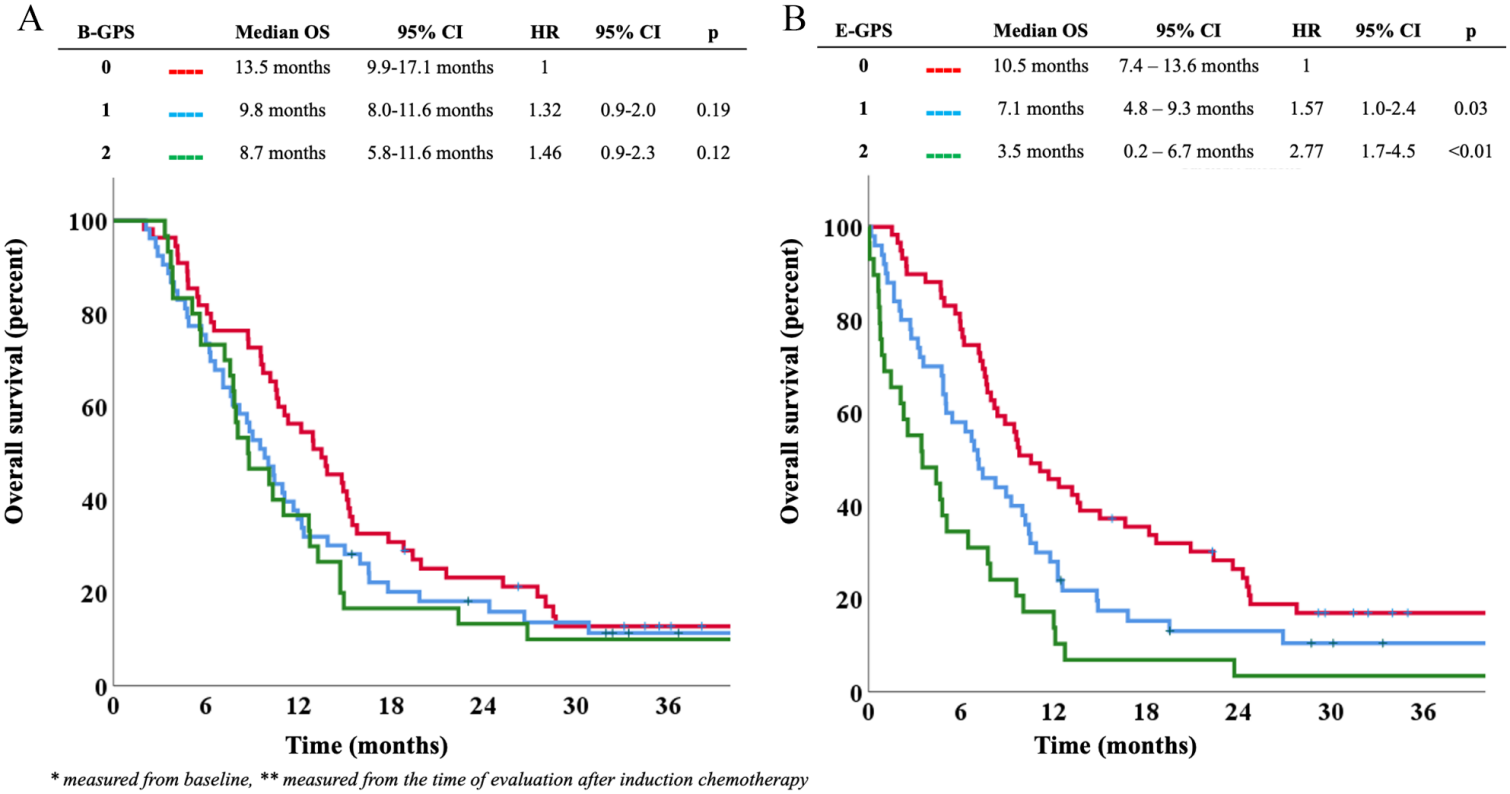
(A) B-GPS and overall survival in the main study cohort.* (B) E-GPS and overall survival in the main study cohort.** B-GPS indicates Glasgow Prognostic Score at baseline; CI, confidence interval; E-GPS, Glasgow Prognostic Score at evaluation; HR, hazard ratio; OS, overall survival. *Measured from baseline. **Measured from the time of evaluation after induction chemotherapy.

Overall, median OS was 10.6 months (95% CI: 9.2-11.9) in the main study cohort (n = 138). Patients with B-GPS 0, 1, and 2 had median OS of 13.5 (95% CI: 9.9-17.1) months, 9.8 (95% CI: 8.0-11.6) months, and 8.7 (95% CI: 5.8-11.6) months, respectively (Figure 2A). There were no statistically significant differences in OS according to B-GPS in univariable or multivariable analyses (Figure 2A and Table 2), nor in a post hoc multivariable analysis in which B-GPS 1 and 2 were pooled and compared with B-GPS 0 (hazard ratio [HR] = 1.27, 95% CI: 0.87-1.88, *P* = .22 in multivariable analysis).

**Table 2.** Univariable and multivariable analyses of B-GPS and overall survival in the main study cohort.^a^

			Univariable analysis			Multivariable analysis		
		n	HR	95% CI	*P*-value	HR	95% CI	*P*-value
GPS at baseline (B-GPS)	0	55	1			1		
	1	53	1.32	0.88-1.98	.19	1.27	0.83-1.93	.27
	2	30	1.46	0.91-2.34	.12	1.29	0.77-2.14	.33
Age (continuous)		138	1.01	0.98-1.03	.60	1.00	0.97-1.03	.91
Sex	Female	70	1			1		
	Male	68	1.14	0.80-1.63	.47	1.14	0.79-1.65	.47
Disease stage	IIIb	8	1			1		
	IV	130	1.04	0.51-2.13	.92	1.03	0.50-2.14	.93
WHO-PS at baseline	0	50	1			1		
	1	80	1.48	1.01-2.18	.05	1.40	0.92-2.12	.12
	2	8	2.67	1.25-5.72	.01	2.53	1.15-5.56	.02

CI, confidence interval; GPS, Glasgow Prognostic Score; HR, hazard ratio; WHO PS, WHO Performance Status.

a Measured from baseline.

Sensitivity survival analysis of all patients with B-GPS measured (n = 208) showed that patients with B-GPS 0, 1, and 2 had median OS of 13.8 (95% CI: 11.1-16.5) months, 7.1 (95% CI: 4.8-9.4) months, and 8.2 (95% CI: 7.1-9.3) months, respectively. For this group, the lower survival in patients with B-GPS 1 compared with B-GPS 0 reached statistical significance (HR = 1.51, 95% CI: 1.1-2.1, *P* = .01) (Supplementary Figure).

In the final sensitivity multivariable survival analysis of the main study cohort (n = 138) adjusting for randomization and whether patients later received immunotherapy, B-GPS was still not a significant prognostic factor (data not shown).

#### E-GPS and survival

Overall, median OS from evaluation after first-line chemotherapy was 7.7 months (95% CI: 6.3-9.2). Patients with E-GPS 0, 1, and 2 had median OS of 10.5 (7.4-13.6) months, 7.1 (4.8-9.3) months, and 3.5 (0.2-6.7) months, respectively. Higher E-GPS was significantly associated with shorter survival time; HR = 1.57 (95% CI: 1.04-2.37, *P* = .03) for E-GPS 1 as compared with E-GPS 0, and HR = 2.77 (95% CI: 1.73-4.45, *P* < .01) for E-GPS 2 as compared with E-GPS 0 (Figure 2B). In the multivariable analysis, the survival difference between E-GPS 2 vs 0 remained statistically significant (*P* < .01), while there was a trend toward a significant difference between E-GPS 1 and 0 patients (*P* = .08) (Table 3).

**Table 3.** Univariable and multivariable analyses of E-GPS and overall survival in the main study cohort.^a^

			Univariable analysis			Multivariable analysis		
		n	HR	95% CI	*P*-value	HR	95% CI	*P*-value
GPS at evaluation (E-GPS)	0	59	1			1		
	1	50	1.57	1.04-2.37	.03	1.47	0.96-2.27	.08
	2	29	2.77	1.73-4.45	<.01	2.11	1.26-3.57	<.01
Age (continuous)		138	1.01	0.98-1.03	.69	1.00	0.97-1.03	.99
Sex	Female	70	1			1		
	Male	68	1.17	0.81-1.66	.42	1.25	0.86-1.83	.24
Disease stage	IIIb	130	1			1		
	IV	8	1.12	0.54-2.29	.76	0.84	0.40-1.77	.65
WHO-PS at evaluation^b^	0	26	1			1		
	1	77	2.38	1.39-4.01	<.01	2.14	1.23-3.73	<.01
	2	21	4.75	2.46-9.18	<.01	4.52	2.31-8.82	<.01
	3	6	26.91	9.61-75.34	<.01	18.52	6.28-54.58	<.01
	4	2	7.75	1.75-34.32	<.01	10.71	2.28-50.17	<.01

CI, confidence interval; GPS, Glasgow Prognostic Score; HR, hazard ratio; WHO PS, WHO Performance Status.

a Measured from the time of evaluation after induction chemotherapy

b Missing in 6 patients.

In the sensitivity multivariable survival analysis adjusting for randomization and whether patients received subsequent immunotherapy, E-GPS but not B-GPS remained a significant prognostic factor (data not shown).

### GPS and response to induction chemotherapy

At evaluation after induction chemotherapy, 38 patients (28%) had partial response (PR), 48 (35%) had stable disease (SD), 48 (35%) had progressive disease (PD), and 4 (3%) were not evaluable (Table 4).

**Table 4.** B-GPS, E-GPS, and change in GPS and response to first-line chemotherapy in the main study cohort.

		GPS at baseline (B-GPS)				GPS at evaluation (E-GPS)				Change in GPS			
	n = 138	B-GPS 0 n = 55	B-GPS 1 n = 53	B-GPS 2 n = 30		E-GPS 0 n = 59	E-GPS 1 n = 50	E-GPS 2 n = 29		Worsened GPS n = 32	Stable GPS n = 73	Improved GPS n = 33	
	n (%)	n (%)	n (%)	n (%)	*P*	n (%)	n (%)	n (%)	*P*	n (%)	n (%)	n (%)	*P*
Partial response	38 (28)	16 (30)	11 (21)	11 (38)		24 (41)	10 (21)	4 (14)		4 (13)	19 (26)	15 (45)	
Stable disease	48 (35)	20 (38)	20 (38)	8 (28)		22 (38)	19 (40)	7 (25)		10 (32)	26 (36)	12 (36)	
Progressive disease	48 (35)	17 (32)	21 (40)	10 (34)	.54	12 (21)	19 (40)	17 (61)	<.01	16 (50)	27 (37)	5 (15)	.01
Not evaluable	4 (3)	2 (1)	1 (1)	1 (1)		1 (1)	2 (1)	1 (1)		2 (6)	1 (1)	1 (3)	

B-GPS was not significantly associated with treatment response (*P* = .54), whereas E-GPS was (*P* < .01). Forty-one percent of patients with E-GPS 0 had achieved a PR, while corresponding numbers among patients with E-GPS 1 and E-GPS 2 were 21% and 14%, respectively. Furthermore, change in GPS was associated with treatment response (*P* = .01). Among patients with improved GPS, 45% had achieved a PR, among those with stable GPS, 26% had a PR, while 13% of those with worse GPS had a PR.

## Discussion

In this study of patients with advanced non-squamous NSCLC, we found that GPS assessed at evaluation after 3 or 4 courses of first-line platinum-doublet chemotherapy (E-GPS) was prognostic for survival, whereas GPS at baseline (B-GPS) was not. Furthermore, patients with a low E-GPS and those with an improved GPS from baseline until evaluation after chemotherapy had higher response rates to chemotherapy than other patients. There was no significant association between B-GPS and response to chemotherapy.

Studies of the prognostic role of GPS in cancer comprise more than 70 000 patients,^7-9^ but only a few have looked at the impact of GPS measured during or after treatment: Three small studies (n = 24-64) reported that elevated GPS measured 3 to 6 weeks after initiation of immune checkpoint inhibitor therapy was associated with poor survival in advanced NSCLC^30,31^ and renal cell carcinoma.^32^ Others have found that elevated GPS after initiation of palliative chemotherapy for colorectal cancer^33^ and after surgery for localized NSCLC^34^ and gastric cancer^15^ was associated with poor prognosis. A study of patients with advanced head and neck cancer found that GPS after concurrent chemoradiotherapy was associated with recurrence free and overall survival, whereas pretreatment GPS was not.^35^ Moreover, elevated modified GPS (mGPS) after neo-adjuvant chemotherapy before surgery for adenocarcinoma of the esophagogastric junction was associated with reduced survival, whereas pre-neo-adjuvant mGPS was not.^16^ Overall, these studies corroborate the results of our study. In contrast, Forrest and colleagues studied patients with inoperable NSCLC treated with chemotherapy^11^ and found that B-GPS was associated with survival, whereas GPS measured 3 to 6 months after inclusion was not. However, only a minority (42%) of patients received active cancer treatment and a minority (38%) had GPS measured during follow-up.

Most previous studies of NSCLC have found B-GPS to be a prognostic factor,^5,6,10^-^13,22,23^ but many studies have pooled B-GPS categories when running the analyses,^6,8^-^10,30,36,37^ which limits the evidence for the prognostic value of each of the 3 different GPS values. Furthermore, most included relatively unselected populations, only subsets of patients received cancer treatment,^6,11^ and many included patients with PS of 3 or 4.^6,11,22,23^ Our main analyses only included patients who completed 3 or 4 courses of chemotherapy, and the majority (94%) had a PS of 0 to 1. Thus, it is possible that B-GPS provides less prognostic information in patients who are considered fit for systemic cancer treatment than in less selected cohorts including cancer patients unfit for palliative chemotherapy, because there might be less variation in prognostic/predictive factors including B-GPS. The potential impact of patient selection might explain why there was a statistically significant survival difference between B-GPS 0 and 1 in our expanded cohort (all 208 with a B-GPS), while this was not the case in the main study cohort.

To the best of our knowledge, no studies have assessed E-GPS and response, but neutrophil-to-lymphocyte ratio (NLR) and CRP after targeted therapy or immune checkpoint blockade were associated with overall response rate in advanced renal cell carcinoma.^32,38,39^ Although treatments, settings, and design are different, these studies support the hypothesis that E-GPS holds more prognostic information than B-GPS because it incorporates the treatment effect. A possible explanation, as hypothesized, is that effective systemic therapy reduces the cancer-induced inflammation. On the contrary, systemic inflammation might reduce the effect of chemotherapy, possibly due to influence on tumor microenvironment.^40^ However, our study was not designed to investigate underlying mechanisms.

The main limitation of our study is the sample size. Furthermore, we cannot rule out that our results might have been influenced by a selection bias. There were only lab values for assessing E-GPS in 138 of the 232 patients included in the trial, and most common reasons for not measuring E-GPS were death, progression, or poor PS. The time frames for measuring CRP and albumin were generous. There was only a trend toward a statistically significant difference between E-GPS 0 and 1 patients in the multivariable survival analysis (*P* = .08). In sensitivity analyses including all patients with B-GPS measured (n = 208), there was a statistically significant difference in survival between patients with B-GPS 0 and 1, possibly indicating that B-GPS have less prognostic value among cancer patients who tolerate palliative chemotherapy than in less selected populations including patients unfit for such therapy. Finally, GPS might also be influenced by malnutrition and side effects from chemotherapy such as nausea and anorexia, and one study shows that patients with a poor B-GPS experience more toxicity from cancer therapy.^13^ Unfortunately, our study was not designed to investigate such complex interactions.

Another limitation of our study is that subsequent treatment differed largely between the participants, especially as immunotherapy was introduced during the study period. However, this is not likely to affect our results, as it would rather be a mediator than a confounder of the association between GPS and OS. And in sensitivity analysis adjusting for group in our original RCT (randomized to pemetrexed maintenance therapy, randomized to observation, or did not meet criteria for randomization) and whether patients received immunotherapy or not, the prognostic value of E-GPS remained stronger than for B-GPS (data not shown). Platinum-doublet chemotherapy alone is no longer standard primary treatment for advanced NSCLC, but our and previous studies have demonstrated associations between E-GPS and response to treatment and survival in patients with several cancers receiving different therapies, indicating that E-GPS reflects treatment effect independently of treatment modality. Finally, CRP and albumin, and thereby GPS, could have been influenced by other factors, ie, infection, inflammation, comorbidity, nutrition, and medication (eg, corticosteroids), but our study was not designed to collect such data. On the contrary, this also applies to most previous studies of GPS.^6,11,23,31^-^37^

The main strength of our study is that we have investigated a relatively uniform patient population. Furthermore, the differences in survival between the 3 E-GPS categories are relatively large and clinically meaningful and might guide clinicians when planning follow-up intervals of patients, when considering maintenance therapy, or switching ongoing treatment. However, the clinical value of E-GPS and how it should be used needs to be further evaluated, ideally in prospective trials.

## Conclusion

To conclude, we found that E-GPS was a stronger prognostic factor than B-GPS and that E-GPS, but not B-GPS, was significantly associated with response to chemotherapy in patients with advanced non-squamous NSCLC.

## Supplemental Material

sj-pptx-1-onc-10.1177_11795549221086578 – Supplemental material for Prognostic Value of Post First-Line Chemotherapy Glasgow Prognostic Score in Advanced Non-Small Cell Lung Cancer Click here for additional data file. Supplemental material, sj-pptx-1-onc-10.1177_11795549221086578 for Prognostic Value of Post First-Line Chemotherapy Glasgow Prognostic Score in Advanced Non-Small Cell Lung Cancer by Kristin Stokke, Marie Søfteland Sandvei, Bjørn Henning Grønberg, Marit Slaaen, Kristin T Killingberg and Tarje O Halvorsen in Clinical Medicine Insights: Oncology

sj-tiff-1-onc-10.1177_11795549221086578 – Supplemental material for Prognostic Value of Post First-Line Chemotherapy Glasgow Prognostic Score in Advanced Non-Small Cell Lung Cancer Click here for additional data file. Supplemental material, sj-tiff-1-onc-10.1177_11795549221086578 for Prognostic Value of Post First-Line Chemotherapy Glasgow Prognostic Score in Advanced Non-Small Cell Lung Cancer by Kristin Stokke, Marie Søfteland Sandvei, Bjørn Henning Grønberg, Marit Slaaen, Kristin T Killingberg and Tarje O Halvorsen in Clinical Medicine Insights: Oncology

## References

[1] Hanahan D Weinberg RA. Hallmarks of cancer: the next generation. Cell. 2011;144:646-674. doi:10.1016/j.cell.2011.02.013. 21376230

[2] Mantovani A Allavena P Sica A Balkwill F. Cancer-related inflammation. Nature. 2008;454:436-444. doi:10.1038/nature07205. 18650914

[3] Coussens LM Werb Z. Inflammation and cancer. Nature. 2002;420:860-867. doi:10.1038/nature01322. 12490959 PMC2803035

[4] Roxburgh CS McMillan DC. Cancer and systemic inflammation: treat the tumour and treat the host. Br J Cancer. 2014;110:1409-1412. doi:10.1038/bjc.2014.90. 24548867 PMC3960633

[5] Forrest LM McMillan DC McArdle CS Angerson WJ Dunlop DJ. Evaluation of cumulative prognostic scores based on the systemic inflammatory response in patients with inoperable non-small-cell lung cancer. Br J Cancer. 2003;89:1028-1030. doi:10.1038/sj.bjc.6601242. 12966420 PMC2376960

[6] Leung EY Scott HR McMillan DC. Clinical utility of the pretreatment Glasgow prognostic score in patients with advanced inoperable non-small cell lung cancer. J Thorac Oncol. 2012;7:655-662. doi:10.1097/JTO.0b013e318244ffe1. 22425914

[7] Dolan RD Laird BJA Horgan PG McMillan DC. The prognostic value of the systemic inflammatory response in randomised clinical trials in cancer: a systematic review. Crit Rev Oncol Hematol. 2018;132:130-137. doi:10.1016/j.critrevonc.2018.09.016. 30447918

[8] Dolan RD Lim J McSorley ST Horgan PG McMillan DC. The role of the systemic inflammatory response in predicting outcomes in patients with operable cancer: systematic review and meta-analysis. Sci Rep. 2017;7:16717. doi:10.1038/s41598-017-16955-5. 29196718 PMC5711862

[9] Dolan RD McSorley ST Horgan PG Laird B McMillan DC. The role of the systemic inflammatory response in predicting outcomes in patients with advanced inoperable cancer: systematic review and meta-analysis. Crit Rev Oncol Hematol. 2017;116:134-146. doi:10.1016/j.critrevonc.2017.06.002. 28693795

[10] Zhu L Chen S Ma S Zhang S. Glasgow prognostic score predicts prognosis of non-small cell lung cancer: a meta-analysis. SpringerPlus. 2016;5:439. doi:10.1186/s40064-016-2093-9. 27104127 PMC4828364

[11] Forrest LM McMillan DC McArdle CS Angerson WJ Dagg K Scott HR. A prospective longitudinal study of performance status, an inflammation-based score (GPS) and survival in patients with inoperable non-small-cell lung cancer. Br J Cancer. 2005;92:1834-1836. doi:10.1038/sj.bjc.6602591. 15870712 PMC2361776

[12] Forrest LM McMillan DC McArdle CS Angerson WJ Dunlop DJ. Comparison of an inflammation-based prognostic score (GPS) with performance status (ECOG) in patients receiving platinum-based chemotherapy for inoperable non-small-cell lung cancer. Br J Cancer. 2004;90:1704-1706. doi:10.1038/sj.bjc.6601789. 15150622 PMC2409737

[13] Gioulbasanis I Pallis A Vlachostergios PJ , et al. The Glasgow Prognostic Score (GPS) predicts toxicity and efficacy in platinum-based treated patients with metastatic lung cancer. Lung Cancer. 2012;77:383-388. doi:10.1016/j.lungcan.2012.04.008. 22551892

[14] Yamamoto Y Matsuyama H Matsumoto H , et al. Prognostic value of risk stratification using blood parameters for nivolumab in Japanese patients with metastatic renal-cell carcinoma. Jpn J Clin Oncol. 2020;50:214-220. doi:10.1093/jjco/hyz168. 31755525

[15] Takeno S Hashimoto T Shibata R , et al. Improvement of high-sensitivity inflammation-based Glasgow prognostic score by gastrectomy is a favorable prognostic factor in patients with gastric cancer. Anticancer Res. 2014;34:5695-5702. 25275076

[16] Jomrich G Hollenstein M John M , et al. The modified Glasgow prognostic score is an independent prognostic indicator in neoadjuvantly treated adenocarcinoma of the esophagogastric junction. Oncotarget. 2018;9:6968-6976. doi:10.18632/oncotarget.24087. 29467943 PMC5805529

[17] Walsh SM Casey S Kennedy R Ravi N Reynolds JV. Does the modified Glasgow Prognostic Score (mGPS) have a prognostic role in esophageal cancer. J Surg Oncol. 2016;113:732-737. doi:10.1002/jso.24225. 27004839

[18] Stotz M Gerger A Eisner F , et al. Increased neutrophil-lymphocyte ratio is a poor prognostic factor in patients with primary operable and inoperable pancreatic cancer. Br J Cancer. 2013;109:416-421. doi:10.1038/bjc.2013.332. 23799847 PMC3721392

[19] Toyokawa T Kubo N Tamura T , et al. The pretreatment Controlling Nutritional Status (CONUT) score is an independent prognostic factor in patients with resectable thoracic esophageal squamous cell carcinoma: results from a retrospective study. BMC Cancer. 2016;16:722. doi:10.1186/s12885-016-2696-0. 27599460 PMC5013653

[20] Ferro M De Cobelli O Buonerba C , et al. Modified Glasgow Prognostic Score is associated with risk of recurrence in bladder cancer patients after radical cystectomy: a multicenter experience. Medicine (Baltimore). 2015;94:e1861. doi:10.1097/MD.0000000000001861. 26496339 PMC4620818

[21] Arigami T Okumura H Matsumoto M , et al. Analysis of the fibrinogen and neutrophil-lymphocyte ratio in esophageal squamous cell carcinoma: a promising blood marker of tumor progression and prognosis. Medicine (Baltimore). 2015;94:e1702. doi:10.1097/MD.0000000000001702. 26496280 PMC4620830

[22] Simmons CP Koinis F Fallon MT , et al. Prognosis in advanced lung cancer—a prospective study examining key clinicopathological factors. Lung Cancer. 2015;88:304-309. doi:10.1016/j.lungcan.2015.03.020. 25870155

[23] Jiang AG Chen HL Lu HY. The relationship between Glasgow Prognostic Score and serum tumor markers in patients with advanced non-small cell lung cancer. BMC Cancer. 2015;15:386. doi:10.1186/s12885-015-1403-x. 25956656 PMC4432878

[24] Neal RD Sun F Emery JD Callister ME. Lung cancer. BMJ. 2019;365:l1725. doi:10.1136/bmj.l1725. 31160279

[25] Proctor MJ Talwar D Balmar SM , et al. The relationship between the presence and site of cancer, an inflammation-based prognostic score and biochemical parameters. Initial results of the Glasgow Inflammation Outcome Study. Br J Cancer. 2010;103:870-876. doi:10.1038/sj.bjc.6605855. 20717110 PMC2966631

[26] Halvorsen TO Stokke K Killingberg KT , et al. Randomized phase III trial comparing switch-maintenance pemetrexed with observation followed by pemetrexed at progression in advanced NSCLC. Acta Oncol. 2020;59:1051-1057. doi:10.1080/0284186x.2020.1778179. 32543258

[27] Eisenhauer EA Therasse P Bogaerts J , et al. New response evaluation criteria in solid tumours: revised RECIST guideline (version 1.1). Eur J Cancer. 2009;45:228-247. doi:10.1016/j.ejca.2008.10.026. 19097774

[28] Reck M Rodriguez-Abreu D Robinson AG , et al. Pembrolizumab versus chemotherapy for PD-L1-positive non-small-cell lung cancer. N Engl J Med. 2016;375:1823-1833. doi:10.1056/NEJMoa1606774. 27718847

[29] Gandhi L Rodriguez-Abreu D Gadgeel S , et al. Pembrolizumab plus chemotherapy in metastatic non-small-cell lung cancer. N Engl J Med. 2018;378:2078-2092. doi:10.1056/NEJMoa1801005. 29658856

[30] Ogura Y Kataoka N Kunimatsu Y , et al. Predictors of survival among Japanese patients receiving first-line chemoimmunotherapy for advanced non-small cell lung cancer. Thorac Cancer. 2021;12:97-105. doi:10.1111/1759-7714.13720. 33124197 PMC7779203

[31] Kasahara N Sunaga N Tsukagoshi Y , et al. Post-treatment Glasgow Prognostic Score predicts efficacy in advanced non-small-cell lung cancer treated with Anti-PD1. Anticancer Res. 2019;39:1455-1461. doi:10.21873/anticanres.13262. 30842182

[32] Noguchi G Nakaigawa N Umemoto S , et al. C-reactive protein at 1 month after treatment of nivolumab as a predictive marker of efficacy in advanced renal cell carcinoma. Cancer Chemother Pharmacol. 2020;86:75-85. doi:10.1007/s00280-020-04088-y. 32537714

[33] Shibutani M Maeda K Nagahara H , et al. Significance of markers of systemic inflammation for predicting survival and chemotherapeutic outcomes and monitoring tumor progression in patients with unresectable metastatic colorectal cancer. Anticancer Res. 2015;35:5037-5046. 26254405

[34] Tomita M Ayabe T Chosa E Nakamura K. Prognostic significance of pre- and postoperative Glasgow prognostic score for patients with non-small cell lung cancer. Anticancer Res. 2014;34:3137-3140. 24922684

[35] Chang PH Wang CH Chen EY , et al. Glasgow prognostic score after concurrent chemoradiotherapy is a prognostic factor in advanced head and neck cancer. Chin J Cancer Res. 2017;29:172-178. doi:10.21147/j.issn.1000-9604.2017.03.02. 28729767 PMC5497203

[36] Chua W Clarke SJ Charles KA. Systemic inflammation and prediction of chemotherapy outcomes in patients receiving docetaxel for advanced cancer. Support Care Cancer. 2012;20:1869-1874. doi:10.1007/s00520-011-1289-3. 21986674

[37] Kasahara N Imai H Naruse I , et al. Glasgow prognostic score predicts efficacy and prognosis in patients with advanced non-small cell lung cancer receiving EGFR-TKI treatment. Thorac Cancer. 2020;11:2188-2195. doi:10.1111/1759-7714.13526. 32495520 PMC7396379

[38] Lalani AA Xie W Martini DJ , et al. Change in neutrophil-to-lymphocyte ratio (NLR) in response to immune checkpoint blockade for metastatic renal cell carcinoma. J Immunother Cancer. 2018;6:5. doi:10.1186/s40425-018-0315-0. PMC5776777 29353553

[39] Templeton AJ Knox JJ Lin X , et al. Change in neutrophil-to-lymphocyte ratio in response to targeted therapy for metastatic renal cell carcinoma as a prognosticator and biomarker of efficacy. Eur Urol. 2016;70:358-364. doi:10.1016/j.eururo.2016.02.033. 26924770

[40] Belli C Trapani D Viale G , et al. Targeting the microenvironment in solid tumors. Cancer Treat Rev. 2018;65:22-32. doi:10.1016/j.ctrv.2018.02.004. 29502037

