# Supplementary material for: Prognostic Value of Post First-Line Chemotherapy Glasgow Prognostic Score in Advanced Non-Small Cell Lung Cancer
Source: Clin Med Insights Oncol. 2022 Mar 22;16:11795549221086578. doi: 10.1177/11795549221086578 (PMC8943446; doi:10.1177/11795549221086578)
Supplement: sj-pptx-1-onc-10.1177_11795549221086578 – Supplemental material for Prognostic Value of Post First-Line Chemotherapy Glasgow Prognostic Score in Advanced Non-Small Cell Lung Cancer [file sj-pptx-1-onc-10.1177_11795549221086578.pptx]

## Slide 1
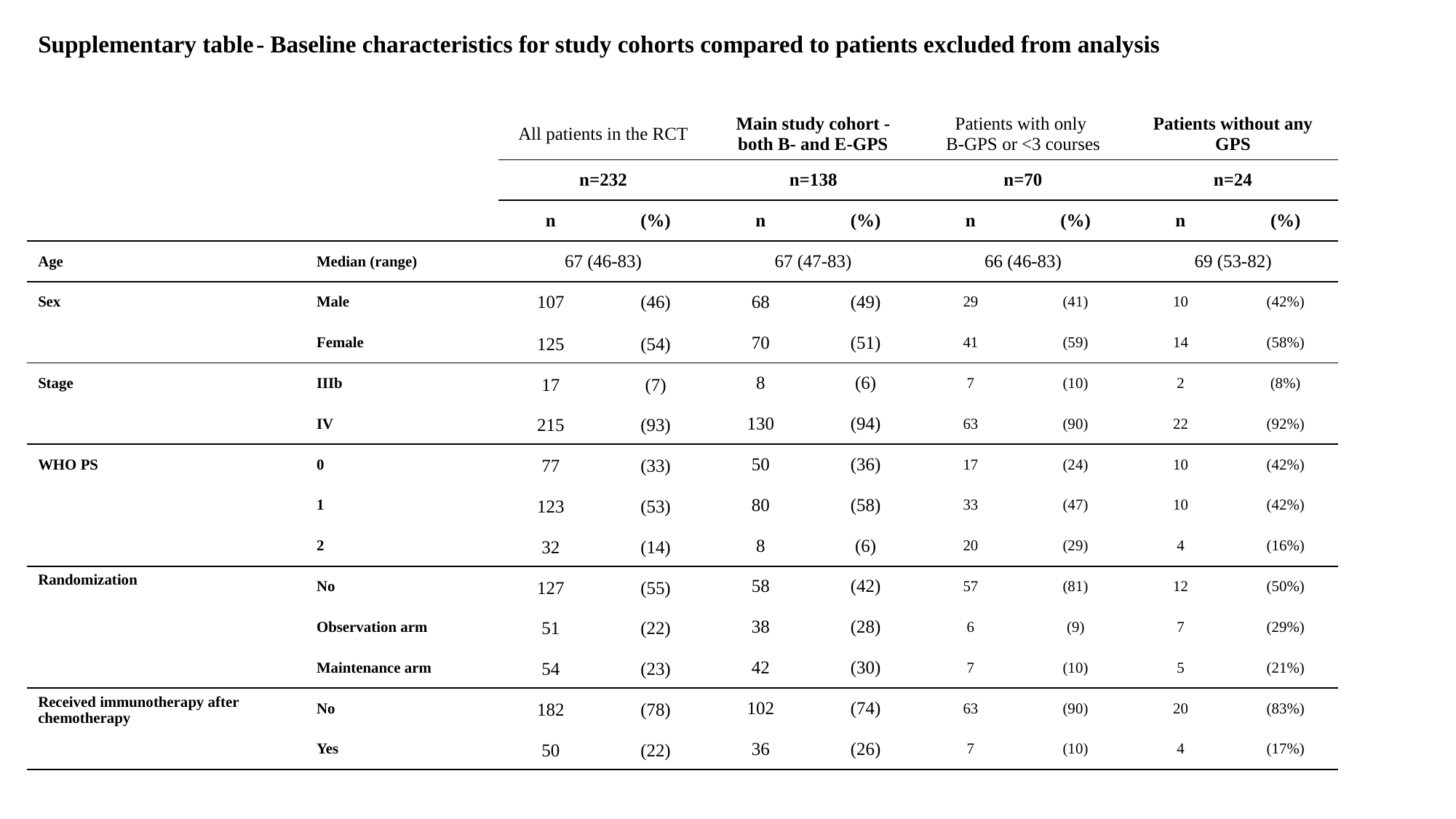

Supplementary table	- Baseline characteristics for study cohorts compared to patients excluded from analysis
| | | All patients in the RCT | | Main study cohort -both B- and E-GPS | | Patients with only B-GPS or <3 courses | | Patients without any GPS | |
| --- | --- | --- | --- | --- | --- | --- | --- | --- | --- |
| | | n=232 | | n=138 | | n=70 | | n=24 | |
| | | n | (%) | n | (%) | n | (%) | n | (%) |
| Age | Median (range) | 67 (46-83) | | 67 (47-83) | | 66 (46-83) | | 69 (53-82) | |
| Sex | Male | 107 | (46) | 68 | (49) | 29 | (41) | 10 | (42%) |
| | Female | 125 | (54) | 70 | (51) | 41 | (59) | 14 | (58%) |
| Stage | IIIb | 17 | (7) | 8 | (6) | 7 | (10) | 2 | (8%) |
| | IV | 215 | (93) | 130 | (94) | 63 | (90) | 22 | (92%) |
| WHO PS | 0 | 77 | (33) | 50 | (36) | 17 | (24) | 10 | (42%) |
| | 1 | 123 | (53) | 80 | (58) | 33 | (47) | 10 | (42%) |
| | 2 | 32 | (14) | 8 | (6) | 20 | (29) | 4 | (16%) |
| Randomization | No | 127 | (55) | 58 | (42) | 57 | (81) | 12 | (50%) |
| | Observation arm | 51 | (22) | 38 | (28) | 6 | (9) | 7 | (29%) |
| | Maintenance arm | 54 | (23) | 42 | (30) | 7 | (10) | 5 | (21%) |
| Received immunotherapy after chemotherapy | No | 182 | (78) | 102 | (74) | 63 | (90) | 20 | (83%) |
| | Yes | 50 | (22) | 36 | (26) | 7 | (10) | 4 | (17%) |
