# Supplementary figures and images for: Prognostic Value of Post First-Line Chemotherapy Glasgow Prognostic Score in Advanced Non-Small Cell Lung Cancer
Source: Clin Med Insights Oncol. 2022 Mar 22;16:11795549221086578. doi: 10.1177/11795549221086578 (PMC8943446; doi:10.1177/11795549221086578)

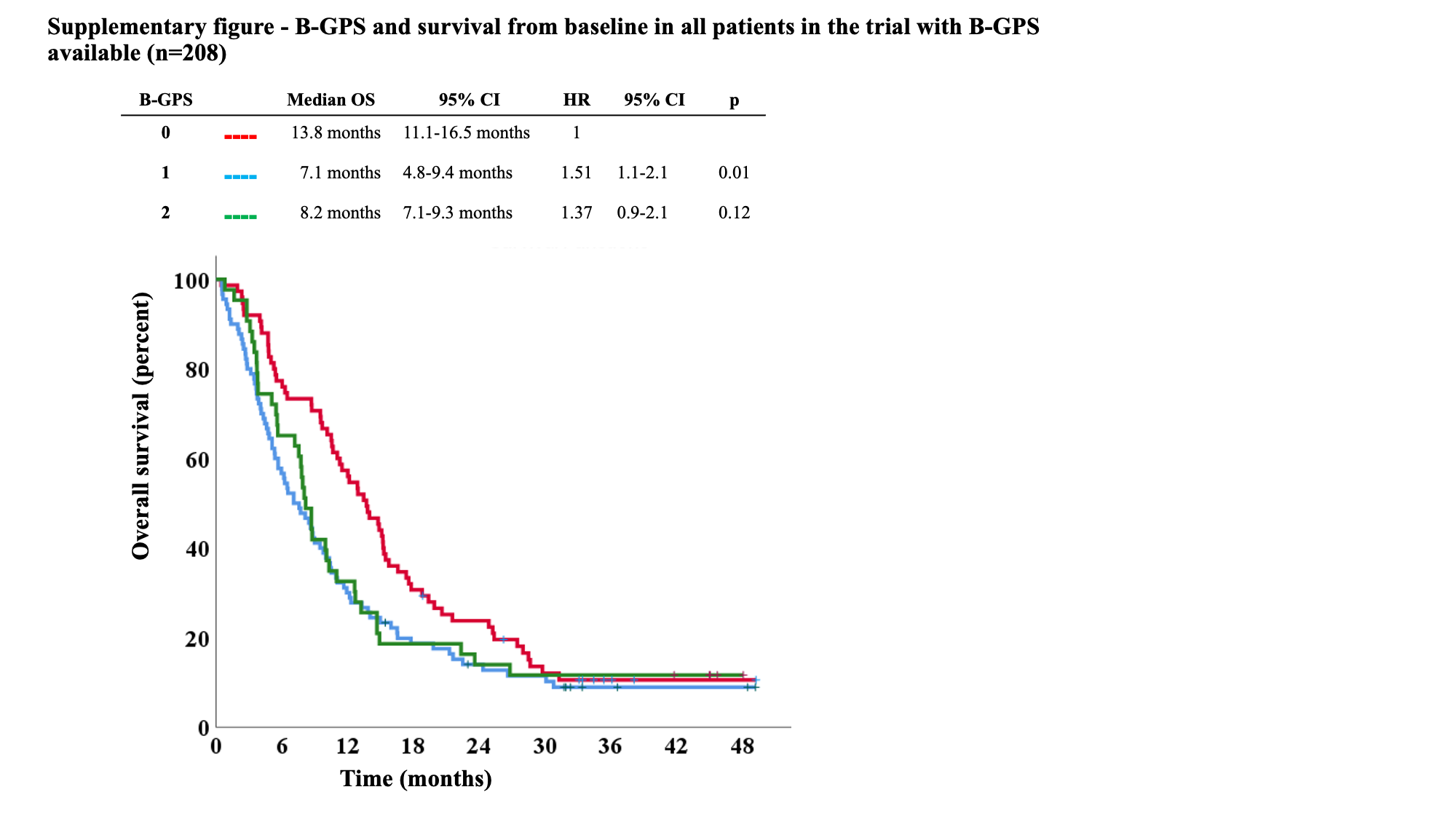

Supplement: sj-tiff-1-onc-10.1177_11795549221086578 – Supplemental material for Prognostic Value of Post First-Line Chemotherapy Glasgow Prognostic Score in Advanced Non-Small Cell Lung Cancer [file sj-tiff-1-onc-10.1177_11795549221086578.tiff]
